# Effect of cervical headgear on dental arch area, shape and interarch dimensions: A randomized study

**DOI:** 10.1007/s00056-020-00264-0

**Published:** 2020-12-03

**Authors:** Toni Heino, Heta Kokko, Ville Vuollo, Pertti Pirttiniemi

**Affiliations:** 1 Research Unit of Oral Health Sciences, Faculty of Medicine, University of Oulu, Oulu, Finland; 2 Medical Research Center, Oulu University Hospital, Oulu, Finland

**Keywords:** Angle class II malocclusion, Space gain, Molars, Growth, Orthodontic treatment, Angle-Klasse-II-Malokklusion, Platzgewinn, Molaren, Wachstum, Kieferorthopädische Behandlung

## Abstract

**Purpose:**

The goal was to study the effects of early cervical headgear treatment on maxillary and mandibular dental arch area, shape and interarch dimensions.

**Methods:**

The total study group comprised 67 children aged 7.6 years (standard deviation 0.3) with Angle class II malocclusion collected between 1992 and 1996. The children were randomly divided into two groups of equal size. In the first group, cervical headgear treatment was started immediately and undertaken for 2 years. The remaining patients served as untreated controls. Dental casts were taken and scanned at the beginning of treatment (T0) and at the 2‑year (T1) and 4‑year follow-up (T2). Three-dimensional landmarks describing the positions of maxillary and mandibular incisors, canines, first and second premolars and first molars were used to calculate and visualize the maxillary and mandibular dental arch area and shape using the polynomial equation *y* *=* *Ax*^6^ *+* *Bx*^2^.

**Results:**

Significant changes in the shape and area of both maxillary and mandibular dental arches were induced with cervical headgear. The headgear increased dental arch area, sagittal dimensions at the mid-sagittal line and transversal dimensions at all of the measured levels in both dental arches compared to the control group.

**Conclusions:**

Cervical headgear is an effective treatment device to gain space in both dental arches. Furthermore, when used as an early phase treatment, relapse is relatively small compared to the gained space.

## Abstract (de)

**Zielsetzung:**

Ziel dieser Studie war es, die Auswirkungen einer frühen zervikalen Headgear-Behandlung auf den Bereich des Ober- und Unterkieferzahnbogens, die Form und die Interbogendimensionen zu untersuchen.

**Methoden:**

Die Studiengruppe umfasste insgesamt 67 Kinder im Alter von 7,6 Jahren (Standardabweichung 0,3) mit Angle-Klasse-II-Malokklusion, die zwischen 1992 und 1996 behandelt wurden. Die Kinder wurden randomisiert in 2 gleich große Gruppen aufgeteilt. In der ersten Gruppe wurde sofort mit der zervikalen Headgear-Behandlung begonnen und sie wurde 2 Jahre lang durchgeführt. Die übrigen Patienten dienten als unbehandelte Kontrolle. Zu Beginn der Behandlung (T0), beim 2‑ (T1) und beim 4‑Jahres-Follow-up (T2) wurden Abdrücke genommen und gescannt. Dreidimensionale Landmarken zur Darstellung der Positionen von Schneidezähnen, Eckzähnen, ersten und zweiten Prämolaren und ersten Molaren im Ober- und Unterkiefer wurden zur Berechnung und Visualisierung des Bereichs und der Form des Zahnbogens des Ober- und Unterkiefers unter Verwendung der Polynomgleichung *y* *=* *Ax*^6^ *+* *Bx*^2^ verwendet.

**Ergebnisse:**

Mit dem zervikalen Headgear wurden signifikante Veränderungen in Form und Fläche sowohl der Ober- als auch der Unterkieferzahnbögen induziert. Das Headgear vergrößerte im Vergleich zur Kontrollgruppe die Zahnbogenfläche, die sagittalen Dimensionen an der mittleren sagittalen Linie und die transversalen Dimensionen auf allen gemessenen Ebenen in beiden Zahnbögen.

**Schlussfolgerungen:**

Das zervikale Headgear ist eine wirksame Behandlungsvorrichtung, um in beiden Zahnbögen Platz zu gewinnen. Wenn sie als Frühphasentherapie eingesetzt wird, ist das Rezidiv im Vergleich zum gewonnenen Platz relativ gering.

## Background

The headgear is a widely used orthodontic appliance for treating Angle class II malocclusion and for distalizing upper molars [1]. It is also a fundamental orthodontic appliance to optimize anchorage for space closure [2]. The headgear should be used 12–14 h per day to achieve sufficient results with treatment [3].

Studies on treatment with cervical headgear combined with fixed appliances have shown an inhibition of forward movement of the maxilla and a posteroinferior redirection of its growth. In addition, downward and backward rotation of the mandible and an improvement of the maxillomandibular relationship was observed together with anterior downward tipping of the palatal plane, palatal tipping of the maxillary incisors, extrusion and distal tipping of the maxillary first molars, reduction of overjet and overbite, and improvement of molar relationship [4]. The headgear also induces molar rotation which results in increased dental arch length. Because of physiological tooth eruption and movement, loss of primary teeth, normal growth and orthodontic movement occur in parallel, it is challenging to define reference points and therefore to reliably study the rotation and movement of the molars [3].

Especially the rotation of molars gains space and lengthens the dental arch. Studies are still controversial since some studies showed only about 1 mm of space gain, whereas some others showed a variation between 2 and 3 mm. According to some studies, space gain can be even more than 3 mm for the total dental arch [3]. Kirjavainen et al. in their study presented children who had severe, moderate or mild maxillary crowding and also mandibular crowding. The use of a headgear resulted in good teeth alignment and enough space gain for all teeth for practically all children [5]. Mäntysaari et al. showed that the space gain in the mandible was approximately half of that in the maxilla [6].

Even though there is long experience and many studies were done with the headgear, the optimal timing of headgear treatment remains controversial. The aim in early treatment is to make later treatment simpler and to avoid extractions [7]. In the study of Kirjavainen et al. 52% of the patients needed phase 2 treatment after cervical headgear therapy mostly because of excess overjet and overbite [5]. The study by Mäntysaari et al. analogously showed that early use of the headgear did not significantly affect overbite or overjet. Tulloch et al. found that there was little difference in the effectiveness of early and later treatment for the correction of class II malocclusion. Headgear treatment was, however, more efficient if started before the eruption of the second maxillary molars [8]. When it comes to treatment’s results and success rate, it seems that expected growth and patient cooperation play a major role [7].

The aim of this study was to determine the effects of early cervical headgear treatment on maxillary and mandibular dental arch area, shape, and interarch dimensions.

## Materials and methods

The material for this study was collected between 1992 and 1996. Informed consent was received from all parents. The existing legislation concerning ethical approval of the study was taken into account and followed in the study. The material comprised 67 children (28 females and 39 males) with a class II malocclusion. The children were randomly divided into two groups. In the first group, Kloehn-type cervical headgear treatment with 6.9–9.8 N force was started immediately or after eruption of the first maxillary molars (mean age 7.3 years, standard deviation [SD] 0.53). The inner bow of the headgear was expanded and constantly held 10 mm wider than the dental arch.

Treatment with the headgear was performed until full class I occlusion was achieved bilaterally. After that, the headgear was worn for retention purpose with less use, until two years of treatment were achieved. In the control group, only minor interceptive procedures were performed during the follow-up period. Fixed appliance treatment, if needed, including extraction of permanent teeth due to crowding, was undertaken after the completion of early treatment. Records were available from the start of the early treatment and at a follow-up after 2 and 4 years. The data are described in more detail in an earlier study by Pirttiniemi et al. [9]. Headgear treatment was undertaken for 2 years from T0 to T1. In this study, we focused on the first 2 years of treatment and the follow-up period of another 2 years.

Dental casts were taken and scanned with 3Shape R700 Dental 3D scanner (3Shape, Copenhagen, Denmark) from both groups at the beginning of treatment T0, at the 2‑year follow-up (T1) and at the 4‑year follow-up (T2). Nine casts had to be excluded from the measurements due to unsuccessful 3D scanning, missing casts, missing teeth, or infraocclusal teeth. Due to these exclusions, the population varies between the groups at T0, T1, and T2. The studied groups at given measurement times are shown in the result tables.

**Fig. 1:**
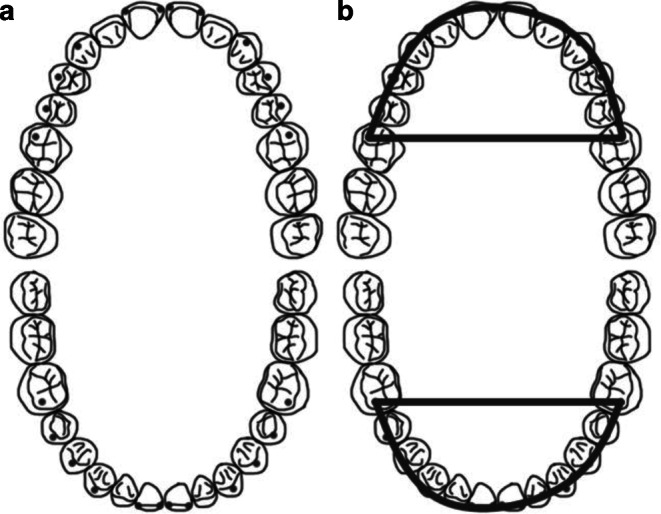
**a** Measurement points (marked with *dots*) of incisors, canines, 1st and 2nd premolars, and 1st molars. **b** Dental arch areas on occlusal surface in both maxillary and mandibular dental arches **a** Messstellen (mit Punkten markiert) von Schneidezähnen, Eckzähnen, ersten und zweiten Prämolaren und ersten Molaren. **b** Zahnbogenbereiche auf der Okklusalfläche in Ober- und Unterkieferzahnbögen

**Fig. 2:**
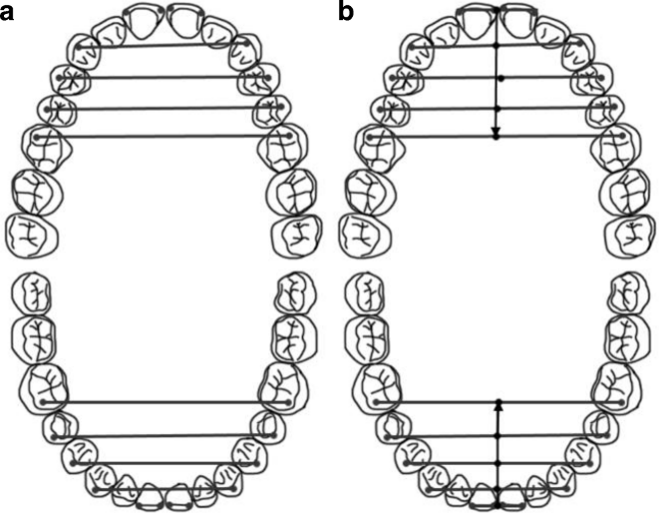
**a** Measurement of the transversal dimension of the dental arch at canine–canine, 1st premolar–1st premolar, 2nd premolar–2nd premolar, 1st molar–1st molar levels in the maxillary and mandibular dental arches. **b** Measurement of the sagittal dimensions of the dental arch between incisor–1st molar, canine–1st molar, 1st premolar–1st molar, 2nd premolar–1st molar **a** Messung der transversalen Dimension des Zahnbogens: Eckzahn-Eckzahn, erster Prämolar - erster Prämolar, zweiter Prämolar - zweiter Prämolar, zweiter Prämolar - zweiter Prämolar, erster Molar - erster Molar im Ober- und Unterkieferzahnbogen. **b** Messung der sagittalen Dimensionen des Zahnbogens: Schneidezahn – erster Molar, Eckzahn – erster Molar, erster Prämolar – erster Molar, zweiter Prämolar – erster Molar

The 3D landmarks describing the positions of maxillary and mandibular incisors, canines, first and second premolars and first molars were used to calculate the characteristics of the maxillary and mandibular dental arches (Fig. 1a). These landmarks were the following: mesial tips of the incisors, buccal cusps of the canines and premolars and mesiobuccal cusps of the first molars. In the case of a missing tooth, the landmark was not placed. After landmarking, the occlusal plane was defined by three landmarks on the maxillary teeth. These landmarks were the following: mesial tips of the central incisors and both mesiobuccal cusps of first molars. Next, all landmarks were projected orthogonally on the occlusion plane. A polynomial equation *y* = *Ax*^6^ + *Bx*^2^ was fitted to the projected landmarks using the least squares method. A and B are coefficients of the polynomial equation and their values are determined by the least squares fitting. This polynomial equation was chosen to model the dental arch form based on the work of Noroozi et al. [10]. The dental arch area was calculated by integrating the fitted polynomial function. The landmarks and dental arch areas are shown in Fig. 1. The projected landmarks were also used to measure both the transversal and sagittal dimensions, i.e., width and depth of the dental arches (Fig. 2). The mean dental arch areas, the width at the first molar level, and the depth between the incisor and the first molar are shown in Table 1. Landmarking, projecting, polynomial fitting, and all the measuring were executed using Rapidform2006 software (INUS Technology, Inc., Seoul, Korea) and all possible processes were automated with a set of in-house VBA (Visual Basic for Applications) subroutines developed for Rapidform. The measured data were analyzed with SPSS statistical software (version 24.0; IBM, Inc., Chicago, IL, USA). These results were compared between the treatment group and the control group and also between the male and female groups. Independent samples t‑test was used to compare the means of the groups after verifying normal distribution of the data.

**Table 1:** Mean dental arch area, mean width of the dental arch at 1st molar level, and mean depth of the dental arch from T0 to T2 Mittlere Zahnbogenfläche, mittlere Breite des Zahnbogens auf Höhe des ersten Molaren und mittlere Tiefe des Zahnbogens von T0 bis T2

**Mean dental arch area**			**Headgear**			**Control**		
			***N***	**Area, mm**^**2**^	**SD, mm**^**2**^	***N***	**Area, mm**^**2**^	**SD, mm**^**2**^
T0	Both	Maxillary	28	1070.1	92.5	34	1052.0	88.3
	Female		12	1031.4	102.3	14	1003.2	55.6
	Male		16	1099.2	75.1	20	1086.1	91.9
	Both	Mandibular	28	851.5	83.0	34	849.0	78.3
	Female		13	819.1	83.6	14	805.0	75.8
	Male		15	879.6	73.9	20	879.8	65.5
T1	Both	Maxillary	29	1189.3	153.9	35	1055.0	112.0
	Female		13	1135.2	195.7	15	998.2	100.9
	Male		16	1233.2	94.7	20	994.5	126.1
	Both	Mandibular	28	910.8	111.6	34	827.2	85.8
	Female		11	867.0	85.9	15	784.3	88.1
	Male		17	939.2	119.2	19	861.1	68.6
T2	Both	Maxillary	28	1202.5	106.5	34	1071.3	140.5
	Female		11	1167.0	100.7	14	1097.6	102.4
	Male		17	1225.5	106.7	20	1125.0	126.5
	Both	Mandibular	26	891.7	110.5	31	809.6	91.6
	Female		11	839.1	105.4	12	767.5	85.9
	Male		15	930.3	100.5	19	836.1	86.8
**Width**			**Headgear**			**Control**		
			***N***	**Width, mm**	**SD, mm**	***N***	**Width, mm**	**SD, mm**
T0	Both	Maxillary	28	49.6	2.3	34	49.0	2.2
	Female		12	48.0	1.6	14	48.5	2.9
	Male		16	50.5	2.2	20	49.4	1.5
	Both	Mandibular	28	44.2	2.1	34	44.4	1.7
	Female		13	43.3	2.2	14	43.4	1.7
	Male		15	45.0	1.6	20	45.1	1.4
T1	Both	Maxillary	29	54.2	2.9	35	49.9	2.0
	Female		13	53.3	3.2	15	49.1	2.3
	Male		16	54.9	2.6	20	50.5	1.5
	Both	Mandibular	28	47.2	2.1	34	44.5	1.7
	Female		11	46.3	1.4	15	43.9	1.6
	Male		17	47.8	2.3	19	45.0	1.5
T2	Both	Maxillary	28	54.2	2.4	34	50.6	2.5
	Female		11	53.1	2.6	14	49.1	2.5
	Male		17	54.7	2.2	20	51.6	1.8
	Both	Mandibular	26	47.2	2.1	31	44.8	2.2
	Female		11	46.1	1.9	12	43.7	2.3
	Male		15	47.8	2.2	19	45.5	1.9
**Depth**			**Headgear**			**Control**		
			***N***	**Depth, mm**	**SD, mm**	***N***	**Depth, mm**	**SD, mm**
T0	Both	Maxillary	28	28.4	1.9	34	28.2	1.9
	Female		12	28.1	2.2	14	27.4	1.3
	Male		16	28.6	1.7	20	28.7	2.1
	Both	Mandibular	28	25.7	1.8	34	25.7	1.4
	Female		13	24.9	2.1	14	25.1	1.2
	Male		15	26.3	1.2	20	26.0	1.4
T1	Both	Maxillary	29	29.9	2.9	35	28.0	2.7
	Female		13	29.0	3.8	15	26.7	2.1
	Male		16	30.7	1.7	20	28.9	2.8
	Both	Mandibular	28	26.2	1.7	34	25.2	1.8
	Female		11	25.5	2.0	15	24.5	1.4
	Male		17	27.0	1.7	19	25.8	1.9
T2	Both	Maxillary	28	30.1	1.4	34	28.1	3.1
	Female		11	29.9	1.4	14	26.5	2.9
	Male		17	30.2	1.5	20	29.0	3.1
	Both	Mandibular	26	26.0	1.8	31	24.7	2.3
	Female		11	25.2	2.0	12	23.6	1.7
	Male		15	26.7	1.5	19	25.4	2.4

*SD* standard deviation, *T0* beginning of treatment, *T1* the 2‑year follow-up, *T2* the 4‑year follow-up

The geometric accuracy of the 3Shape R700 scanner was verified to be 0.02 mm. Previous studies have proven that landmarking dental 3D models is reliable [11] and the measurements on 3D surfaces have excellent accuracy [12].

## Results

### Changes in dental arch area

Results show that the mean increase in maxillary dental arch area from T0 to T1 in the headgear group was 10.0% (95% confidence interval [CI] 5.6–14.4, *p* < 0.001) and that there was no change in the control group (0.0%, 95% CI −2.7 to 2.6, *p* < 0.001). The mean increase in the mandibular dental arch area from T0 to T1 in the headgear group was 5.5% (95% CI 2.6–8.4, *p* < 0.001) and there was a 2.4% (95% CI −4.7 to −0.1, *p* < 0.001) reduction in the control group in the same time period. Therefore, there was a difference of 7.9 percentage points between the headgear and the control group with respect to the change of the mandibular dental arch area at T1. From T0 to T2 the maxillary area mean change in the headgear group was 12.3% (95% CI 8.3–16.3, *p* < 0.001) and the mandibular area mean change was 4.2% (95% CI 0.2–8.3, *p* < 0.001), respectively. In the control group, the change of the maxillary dental arch area was 1.3% (95% CI −2.7 to 5.2, *p* < 0.001) and −5.6% (95% CI −8.6 to −2.6, *p* < 0.001) in the mandibular dental arch area (Table 2). The difference between the male and female groups was also studied and the results showed that there was no significant difference between the genders.

**Table 2:** Changes in mean mandibular and maxillary dental arch area at T0, T1, and T2 in the headgear and control groups Veränderungen der mittleren Unterkiefer- und Oberkieferzahnbogenfläche bei T0, T1 und T2 in den Headgear- und Kontrollgruppen

	**Headgear**				**Control**				
**T0–T1**	***N***	**Dental arch area mean change, %**	**SD, %**	**95% CI**	***N***	**Dental arch area mean change, %**	**SD, %**	**95% CI**	***p***
M + F, maxillary	26	10	11	5.6–14.4	34	0	7.6	−2.7 to 2.6	*<0.001*
M + F, mandibular	25	5.5	7	2.6–8.4	33	−2.4	6.6	−4.7 to −0.1	*<0.001*
Male, maxillary	14	11.8	9.3	6.4–17.2	20	1.1	5.7	−1.5 to 3.8	*<0.001*
Male, mandibular	14	4.8	6.6	1.0–8.6	19	−1.8	6.6	−5.0 to 1.4	*0.008*
Female, maxillary	12	7.9	12.7	−0.2 to 16	14	−1.6	9.8	−7.3 to 4.0	*0.041*
Female, mandibular	11	6.4	7.7	1.2–11.6	14	−3.2	6.7	−7.1 to 0.7	*0.003*
**T0–T2**	***N***	**Dental arch area mean change, %**	**SD, %**	**95% CI**	***N***	**Dental arch area mean change, %**	**SD, %**	**95% CI**	***p***
M + F, maxillary	25	12.3	9.7	8.3–16.3	33	1.3	11.1	−2.7 to 5.2	*<0.001*
M + F, mandibular	24	4.2	9.6	0.2–8.3	30	−5.6	8	−8.6 to −2.6	*<0.001*
Male, maxillary	15	12	10.4	6.3–17.8	20	3.6	8.4	−0.3 to 7.5	*0.012*
Male, mandibular	13	5.8	9	−0.4 to 11.2	19	−4.4	8.7	−8.6 to −0.2	*0.003*
Female, maxillary	10	12.6	9.2	6.0–19.2	13	−2.3	14	−10.8 to 6.2	*0.008*
Female, mandibular	11	2.4	10.4	−4.6 to 9.4	11	−7.7	6.5	−12.1 to −3.3	*0.013*

*SD* standard deviation, *T0* beginning of treatment, *T1* 2‑year follow-up, *T2* 4‑year follow-up, *M* male, *F* female, *95% CI* 95% confidence interval

**Fig. 3:**
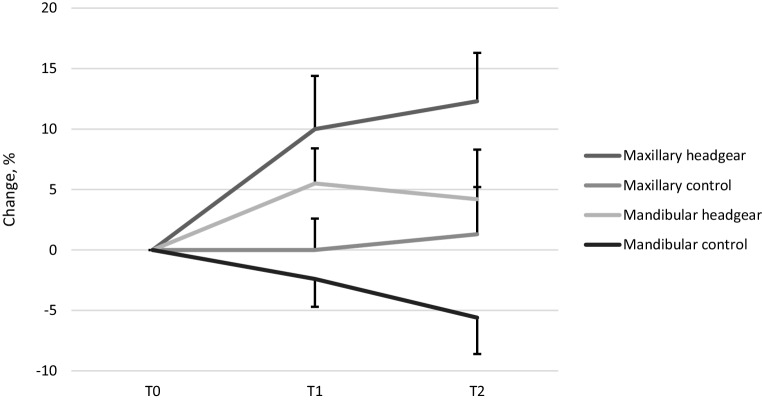
Maxillary and mandibular dental arch area change at T0, T1 and T2 with 95% confidence interval. Only positive or negative error bars are shown to avoid overlapping of the bars Veränderung des Zahnbogenbereichs im Ober- und Unterkiefer bei T0, T1 und T2 mit 95 %-Konfidenzintervall. Um Überlappungen der Balken zu vermeiden, werden nur positive bzw. negative Fehlerbalken angezeigt

**Fig. 4:**
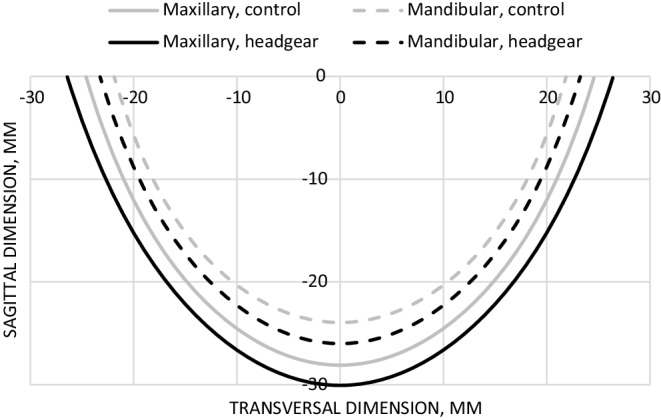
Mean dental arches of both sexes combined at T2. Dental arch area calculated using the polynomial equation *y* = *Ax*^6^ + *Bx*^2^. Maxillary and mandibular dental arches of the headgear group are larger compared to the control group Mittlere Zahnbögen von Patienten beider Geschlechter kombiniert bei T2. Zahnbogenfläche berechnet mit der Polynomgleichung *y* = *Ax*^6^ + *Bx*^2^. Ober- und Unterkieferzahnbögen der Headgear-Gruppe sind größer als in der Kontrollgruppe

The differences between the maxillary and mandibular dental arch areas from T0 to T1 and T2 are shown in Table 3 and in Fig. 3. Dental arch shape and difference in size at T2 are shown in Fig. 4. At the beginning of the study, no significant difference was found between the headgear group and the control group. At T1, the difference between the maxillary and mandibular dental arch areas was 34.9% (95% CI 29.8–40.0) in the headgear group and 27.5% (95% CI 24.0–31.0) in the control group (*p* = 0.014). At T2, the difference between the maxillary and mandibular dental arch areas was 36.3% (95% CI 31.3–41.2) in the headgear group and 32.1% (95% CI 27.1–37.1) in the control group.

**Table 3:** Differences between mean mandibular and maxillary dental arch areas at T0, T1, T2, and between headgear and control groups Unterschiede zwischen mittleren Unterkiefer- und Oberkieferzahnbogenbereichen bei T0, T1, T2 und zwischen Headgear- und Kontrollgruppen

	Headgear				Control				
T0–T2	*N*	Max/Mand mean difference, %	SD, %	95% CI	*N*	Max/Mand mean difference, %	SD, %	95% CI	*p*
T0	27	26.0	10.7	21.8–30.2	34	24.5	11.7	20.5–28.6	0.619
T1	26	34.9	12.6	29.8–40.0	34	27.5	10.1	24.0–31.0	*0.014*
T2	26	36.3	12.2	31.3–41.2	31	32.1	13.6	27.1–37.1	0.234

*95% CI* 95% confidence interval, *T0* beginning of treatment, *T1* 2‑year follow-up, *T2* 4‑year follow-up, *M* male, *F* female, *Max* maxillary, *Mand* mandibular, *SD* standard deviation

### Changes in dental arch transversal dimensions

All the transversal dimensions in the headgear and the control group from T0 to T1 and T2 were significantly increased (*p* < 0.05) except for the transversal dimension at the canine to canine level (Table 4). In the headgear group, the transversal dimensions of the dental arch increased by an average of 9.8% (*p* < 0.001) from T0 to T1 and 11.3% (*p* < 0.001) from T0 to T2 in the maxilla. In the mandible, the increases were 6.5% (*p* < 0.001) and 7.1% (*p* < 0.05), respectively. In the control group, the increases were 2.2% (*p* < 0.001) from T0 to T1 and 4.4% (*p* < 0.001) from T0 to T2 in the maxilla and 0.5% (*p* < 0.001) from T0 to T1 and 2.5% (*p* < 0.05) from T0 to T2 in the mandible. The transversal dimension at the first premolar level in the maxillary dental arch increased by 10.4% (95% CI 7.9–12.8) from T0 to T1 in the headgear group compared to 1.9% (95% CI 0.0–3.8) increase in the control group showing a difference of 8.5 percentage points between the groups (*p* < 0.001). The transversal dimension at the first molar level in the maxillary dental arch increased by 9.5% (95% CI 7.2–11.7) in the headgear group compared to 1.7% (95% CI 0.3–3.1) increase in the control group from T0 to T1 showing a difference of 7.8 percentage points between the groups (*p* < 0.001). From T0 to T2 the increase in transversal dimension in the headgear group was in the maxilla 6.9 percentage points and in the mandible 4.6 percentage points larger on average in comparison to the control group.

**Table 4:** Mean transversal dimension of the dental arch compared at canine–canine, 1st premolar–1st premolar, 2nd premolar–2nd premolar, 1st molar–1st molar level between headgear and control groups at T0–T1 and at T0–T2 Mittlere transversale Dimension des Zahnbogens im Vergleich zwischen Headgear- und Kontrollgruppe bei T0-T1 und bei T0-T2 auf der Höhe von Eckzahn - Eckzahn, 1. Prämolar - 1. Prämolar, 2. Prämolar - 2 Prämolar, 1. Molar - 1. Molar

	Headgear					Control					
**Maxillary, T0–T1**	***N***	**Transversal mean change**		**SD, %**	**95% CI**	***N***	**Transversal mean change**		**SD, %**	**95% CI**	***p***
		**%**	**Mm**				**%**	**Mm**			
Canine	20	11	3.4	7.3	7.6–14.4	21	3	0.9	6.4	0.1–6.0	*0.001*
1st Premolar	23	10.4	3.9	5.7	7.9–12.8	26	1.9	0.7	4.8	0.0–3.8	*<0.001*
2nd Premolar	25	8.1	3.5	4.6	6.2–10.0	31	2	0.8	4.4	0.4–3.6	*<0.001*
1st Molar	26	9.5	4.6	5.6	7.2–11.7	34	1.7	0.8	4	0.3–3.1	*<0.001*
**Maxillary, T0–T2**	***N***	**Transversal mean change**		**SD, %**	**95% CI**	***N***	**Transversal mean change**		**SD, %**	**95% CI**	***p***
		**%**	**Mm**				**%**	**Mm**			
Canine	21	11.3	3.5	9.1	7.1–15.4	21	7.3	2.3	7	4.1–10.5	***0.124***
1st Premolar	21	13.9	5.3	6.8	10.8–17.0	27	5.7	2.1	5.7	3.5–8.0	***<0.001***
2nd Premolar	21	10.5	4.4	4.7	8.4–12.7	28	4.3	1.8	5	2.3–6.2	***<0.001***
1st Molar	24	9.6	4.5	4.4	7.8–11.5	33	3.2	1.5	4.8	1.5–4.9	***<0.001***
**Mandibular, T0–T1**	***N***	**Transversal mean change**		**SD, %**	**95% CI**	***N***	**Transversal mean change**		**SD, %**	**95% CI**	***p***
		**%**	**Mm**				**%**	**Mm**			
Canine	20	6.8	1.7	6.3	3.8–9.7	16	3.2	0.8	5	0.5–5.9	0.074
1st Premolar	21	6.5	2.1	6.1	3.7–9.3	29	0.6	0.2	4.7	−1.2 to 2.3	*<0.001*
2nd Premolar	25	6.2	2.4	2.8	5.1–7.4	31	0.4	0.2	2.1	−0.4 to 1.1	*<0.001*
1st Molar	25	6.8	3.0	3.5	5.3–8.2	33	0.6	0.3	2.3	−0.2 to 1.4	*<0.001*
**Mandibular, T0–T2**	***N***	**Transversal mean change**		**SD, %**	**95% CI**	***N***	**Transversal mean change**		**SD, %**	**95% CI**	***p***
		**%**	**Mm**				**%**	**Mm**			
Canine	21	4.6	1.1	8.2	0.8–8.3	24	5.2	1.3	7.3	2.1–8.3	0.797
1st Premolar	22	8.7	2.9	7.3	5.5–12.0	26	4.7	1.5	4.9	2.7–6.7	*0.029*
2nd Premolar	23	6.4	2.6	4	4.7–8.1	28	2.2	0.8	4.7	0.4–4.0	*0.001*
1st Molar	25	6.3	3.0	3.7	4.8–7.9	30	0.7	0.3	4.3	−0.9 to 2.3	*<0.001*

*SD* standard deviation,* 95% CI* 95% confidence interval, *T0* beginning of treatment, *T1* 2‑year follow-up, *T2* 4‑year follow-up

### Changes in dental arch sagittal dimensions

The mean sagittal increase was larger in both the maxillary and mandibular dental arches and also from T0 to T1 and T2 in the headgear group in comparison to the control group (Table 5). The mean maxillary sagittal dimension between the incisors and the first molars increased by 4.3% (95% CI 1.1–7.4) in the headgear group and decreased by 1.1% (95% CI −2.8 to 0.6) in the control group at T1 when compared to the starting point at T0 (*p* = 0.002). At T2, the increase was 5.3% (95% CI 2.8–7.9) in the headgear group compared to a decrease of −0.8% (95% CI −3.7 to 2.1) in the control group (*p* = 0.003). The mean mandibular sagittal dimension between the incisors and the first molars increased by 1.9% (95% CI 0.5–3.3) in the headgear group and decreased by 1.8% (95% CI −3.3 to −0.4) in the control group from T0 to T1 (*p* = 0.001). At T2, the increase was 0.5% (95% CI −1.6 to 2.6) in the headgear group compared to a decrease of 4.4% (95% CI −7.1 to −1.6) in the control group (*p* = 0.009).

**Table 5:** Mean changes in sagittal dimension between incisor–1st molar level between headgear and control groups from T0–T1 and from T0–T2 Mittlere Veränderungen in der Sagittalebene zwischen Headgear- und Kontrollgruppen von T0-T1 und von T0-T2, Höhe Eckzahn bis erster Molar

	Headgear					Control					
Dental arch, time period	*N*	Sagittal mean change		SD, %	95% CI	*N*	Sagittal mean change		SD, %	95% CI	*p*
		%	Mm				%	Mm			
Maxillary, T0–T1	26	4.3	1.2	7.8	1.1–7.4	34	−1.1	−0.3	4.9	−2.8 to 0.6	*0.002*
Maxillary, T0–T2	25	5.3	1.4	6.2	2.8–7.9	33	−0.8	−0.2	8.1	−3.7 to 2.1	*0.003*
Mandibular, T0–T1	25	1.9	0.5	3.4	0.5–3.3	33	−1.8	−0.5	4.1	−3.3 to −0.4	*<0.001*
Mandibular, T0–T2	24	0.5	0.1	5.1	−1.6 to 2.6	30	−4.4	−1.1	7.4	−7.1 to −1.6	*0.009*

*SD* standard deviation,* 95% CI* 95% confidence interval, *T0* beginning of treatment, *T1* at 2‑year follow-up, *T2* at the 4‑year follow-up

## Discussion

It has been shown that by using the polynomial equation *y* *=* *Ax*^6^ *+* *Bx*^2^ it is possible to study the shape and area of the dental arch. The use of this equation has earlier been shown to be reliable to analyze dental arch form and size [10]. Both the maxillary and mandibular dental arches of the headgear group showed a significant increase in area compared to the control group. From T0 to T2 the maxillary area mean change in the headgear group was 12.3% and the mandibular area mean change was 4.2% respectively. In the control group, the maxillary dental arch area increased only by 1.3% and the mandibular dental arch area decreased by 5.6%. More increase can be seen in both the maxillary and mandibular dental arch areas in the headgear group compared to the control group. McDonald et al. [3] and Mäntysaari et al. [6] have expressed similar results in their studies. The work of Mäntysaari et al. [6] was based on the same material as the present study and they showed that the space gain in the mandible was approximately half of the space gain in the maxilla which is confirmed by the results of our study. However, in the former studies the dental arch area was not studied. In both the headgear and control groups, a slight decrease in mandibular dental arch area was seen from T1 to T2. However, the decrease was lower in the headgear group. The decrease in area might be explained by some relapse caused by insufficient use of the retention appliances, which would have prevented or decreased dental relapse [13]. There was a difference in increase between the mean mandibular and maxillary dental arch areas from T0 to T2 in both groups. The change was greater in the headgear group. This is a normal outcome of headgear treatment because the force of the headgear is transmitted directly to the maxilla. In the maxilla, treating crowding with the headgear alone is successful in many cases. But in the mandible, headgear treatment may not be enough for treating severe crowding.

The increase in transversal dimension in the headgear group appeared higher than in the control group at all measured levels, in both dental arches. In the headgear group the dental arch transversal dimension increased by an average of 11.3% from T0 to T2 in the maxilla. In the control group, the average increase was only 4.4% in the maxilla. The mean sagittal dimension increase was also greater in both the maxillary and mandibular dental arch and from T0 to T1 and from T0 to T2 in the headgear group in comparison with the control group. Freitas et al. [4] have expressed the view that the increased sagittal dimension is achieved by distalizing the first molars and inhibiting the forward growth of the maxilla. However, with respect to this measurement, only the incisor to the first molar sagittal dimension was statistically significant. Thus, no conclusions can be made regarding the sagittal relations for other measured teeth.

The polynomial equation can be used to visualize the dental arches. In the headgear group, both the maxillary and mandibular dental arches were larger compared to the control group. Even though the mean dental arch area can be calculated from the polynomial equation, it cannot be used to accurately calculate sagittal or transversal dimensions of the dental arch at any specific levels as it is only an estimation of the overall dental arch. Therefore, specific measurement points were used to calculate the sagittal and transversal dimensions in this study.

In the present study, the headgear treatment was initiated earlier than in most other studies [14–16]. It is interesting that in the study of Julku et al. [17] where early or later headgear treatment was compared, a larger effect was achieved in the dental arches in the early treatment group. When the treatment effect was compared between the genders, a larger effect was seen in boys. One possible explanation for this could be that boys and girls were in a different maturation phase [18]. When maximal space gain is the goal of the headgear treatment, early onset of treatment could be beneficial, especially before eruption of the second molars.

As can be seen from the results, the headgear is an effective treatment method for gaining space in the dental arches. The headgear increased dental arch area in the maxilla and in the mandible, even though the force is transmitted directly to maxilla. In mild cases, the space gain in the mandible may be enough and no other treatment is required after headgear treatment. The results also show that there was little to no relapse in the maxillary transversal dimension after headgear treatment and the dental arch area remained stable or even continued to increase after headgear treatment. In the mandible, however, relapse could be seen after headgear treatment. Even though some relapse occurred in the mandible in both the headgear group and the control group, the difference between these groups remained significant.

## Conclusions

This study supports using the polynomial equation *y* *=* *Ax*^6^ *+* *Bx*^2^ to study and visualize changes in shape and area of the dental arch. The results show that the headgear is an effective treatment for gaining space in the dental arches. In addition, the results show that when used as an early phase treatment, the relapse is relatively small compared to the gained space.
